# Resistance to Ultraviolet Aging of Nano-SiO_2_ and Rubber Powder Compound Modified Asphalt

**DOI:** 10.3390/ma13225067

**Published:** 2020-11-10

**Authors:** Guoping Qian, Changdong Yang, Haidong Huang, Xiangbing Gong, Huanan Yu

**Affiliations:** State Engineering Laboratory of Highway Maintenance Technology, Changsha University of Science and Technology, Changsha 410114, China; guopingqian@csust.edu.cn (G.Q.); ycd960203@163.com (C.Y.); haidong@163.com (H.H.); huanan.yu@csust.edu.cn (H.Y.)

**Keywords:** compound modification, ultraviolet aging, rheological properties, nano-SiO_2_, rubber powder

## Abstract

Ultraviolet (UV) aging degrades the life span of asphalt pavement, nanomaterials used as modifiers exhibit good shielding function on UV light, but generally degrade the low-temperature property of asphalt, a compound modification was found to be a solution. In this study, nano-SiO_2_ and rubber powder were blended together with base asphalt to prepare compound modified asphalt. Compound modified asphalt with different blending dosages were subjected to UV light via a self-made UV aging simulation chamber. Basic performance tests and rheological tests were conducted including the UV aging influence. An optimum compound ratio was finally recommended based on the goal to remove the adverse effect of nano-SiO_2_ on the thermal cracking. Results show that the anti-UV aging property of asphalt is improved obviously due to the blocking function of nano-SiO_2_ and carbon black in rubber powder, and the enhancing effect of nano-SiO_2_ is found to be the most significant.

## 1. Introduction

Asphalt pavement is directly exposed to external factors during its service period, such as sunlight, oxygen, water, heat, and driving load. Factors generally lead to properties degradation of paving material and service issues of pavement performance [1,2,3,4]. Among all short-term and long-term damages, aging commonly occurs at the surface layer and brings several distresses as aging develops [5]. In general, aging type of asphalt materials is divided into thermal oxygen aging and photooxidative aging. The effect of aging on the performance of asphalt is very huge, aging will make the asphalt hard and brittle, and then easy cracking degrades the road service capacity [6]. In some areas with strong ultraviolet (UV) light, UV aging becomes a predominant fact and seriously affects the rheological properties of asphalt and life span of asphalt pavement [7,8]. However, in current aging resistance evaluation specification, only thermal oxygen aging is included except for UV aging. Qian et al. [5,7] have been studying the UV aging degradation via developing a UV light apparatus to simulate the UV aging in pavement surface layer. Therefore, the UV light can be effectively included in laboratory tests to evaluate resistance to UV aging of asphalt materials.

In recent years, nanomaterials have been widely used as modifiers to enhance materials’ properties. Cheraghian et al. [9] utilized nanomaterials to improve the rheological and fluid loss properties of water-based drilling fluids. In asphalt materials, nanomaterials not only improve the properties of asphalt but also greatly improve the anti-UV aging performance of asphalt [10,11,12]. Asphalt modified with nanomaterials and other chemical modifiers shows strong resistance against high-temperature rutting and low-temperature cracking [13]. Fini et al. [14] studied the effect of nano-SiO_2_ on rheological and aging properties of asphalt, results show that nano-SiO_2_ improves the fundamental and anti-aging properties of asphalt. Cheraghian et al. [15] used dynamic shear rheometer (DSR) and Fourier transform infrared (FT-IR) spectroscopy to study modification method by adding clay and nano-SiO_2_. Results present that clay and nano-SiO_2_ can improve the anti-UV aging performance of asphalt. Yao et al. [16] explored the rheological properties and molecular structure of nano-SiO_2_ modified asphalt, scholars conducted aging tests on nano-SiO_2_ modified asphalt. It was found that the viscosity decreased slightly, and the low-temperature performance decreased, but the anti-aging property significantly improved. Shafabakhsh et al. [17] prepared modified asphalt mixtures with different nano-TiO_2_ contents, it was told that nano-TiO_2_ leaded great enhancement on permanent deformation resistance and fatigue life of asphalt mixtures. Related studies [12,18,19] exhibit that the nano-SiO_2_ and nano-TiO_2_ can improve the anti-aging properties of asphalt but may have a negative effect on the low-temperature properties of asphalt. 

Chemical additives have been used to improve the road performance of base asphalt for many decades. The low-temperature cracking property and high-temperature stability of modified asphalt are superior to non-modified asphalt [18,20]. Additionally, application of waste rubber on the road can solve the problem of disposal of used tires and environment issues [21]. Li et al. [22] conducted thermal oxygen aging tests based on the analysis of the composition and structure, the thermal oxygen aging mechanism of rubber powder modified asphalt was discussed in this study, results indicate that rubber powder modified asphalt has an excellent anti-aging performance. Pang et al. [23] blended an anti-UV aging double hydroxide with rubber powder modified asphalt to study the anti-aging characteristic of compound modified asphalt, the short-term and long-term aging data imply that the modified asphalt has better UV aging resistance. Xiao et al. [24] carried out high-performance gel chromatography (HP-GPC) test on long-term aging samples of rubber powder modified asphalt, base asphalt and styrene-butadiene-styrene (SBS) modified asphalt to analyze the molecular distribution (MSD) of asphalt subjected to aging, it was found that the macromolecules of rubber asphalt showed the least increase in proportion, rubber powder is proved to improve the low-temperature cracking and aging resistance of asphalt.

Currently, nano-SiO_2_ has been widely applied to make modified asphalt. Different sources, different dosages, and different blending methods of modified asphalt will affect the rheological properties and aging properties of asphalt [25]. Nano-SiO_2_ presents a special silane tetrahedral structure known as agglomeration [26,27]. Generally, the high-temperature performance of nano-SiO_2_ modified asphalt is better than base asphalt, and the high-temperature performance of modified asphalt is more excellent with the increase of nano-SiO_2_ content. However, the higher nano-SiO_2_ amount does not mean better performance [28]. Nano-SiO_2_ was proved to affect the low-temperature performance of the modified asphalt [29,30,31]. As a waste material, rubber powder modified asphalt shows successful application on asphalt modification. The amount of waste rubber powder in rubber asphalt is generally between 5% and 30% based on related studies [32,33]. Results tell that 20% rubber powder improves the high and low-temperature properties, anti-aging properties, and temperature sensitivity of asphalt [34,35]. However, the fatigue life of the rubber powder modified asphalt mixture increased with the crumb rubber content, but only to a certain percent before it started to decrease again [36].

Therefore, to eliminate the effect of nano-SiO_2_ on the low-temperature property of asphalt, this study selects the compound modification method by using rubber powder as a topic. In order to evaluate the comprehensive properties of asphalt used in strong UV sunlight regions, this study mainly aims at anti-UV aging capacity as well as the fundamental performances of nano-SiO_2_ and rubber powder compound modified asphalt especially the low-temperature property. It firstly conducted property evaluation of asphalt under different compounding ratios before aging by conventional performance tests and rheological property tests. Then, a self-made UV environment device was used to simulate the UV aging, and the anti-UV aging property of modified asphalt with different compound ratios were analyzed. The main objective of this study is to explore the optimum compounding ratio of nano-SiO_2_ and rubber powder, the predominant goal is to remove the disadvantage of nano-SiO_2_ on low-temperature cracking property without degradation of other performances. The other objective is to assess the UV aging resistance of compound modified asphalt and distinguish the internal function of rubber powder and nano-SiO_2_.

## 2. Materials and Methods

### 2.1. Physical Properties of Raw Materials

This study selected a type of nano-SiO_2_ produced by Zhongkexing Co., Ltd. (Wuhan, China), and its physical properties are shown in Table 1.

Rubber powder is a kind of waste rubber powder produced by Zhejiang Green Ring Co., Ltd. (Zhejiang, China), and its physical properties are shown in Table 2.

The base asphalt is 70#A grade petroleum asphalt produced by Sinopec Maoming Branch (Guangdong, China). The physical properties are shown in Table 3.

All raw materials’ properties meet the industrial specification requirement of China.

### 2.2. Preparation of Modified Asphalt

According to the literature review, the dosage range of nano-SiO_2_ is 3~5%, the amount of 80# mesh rubber powder used in this study is chosen to be 12~20% The design of the compound modification is controlled by the control variable method. For the convenience to label the experimental group, “3% nano-SiO_2_” is abbreviated as “3S”, “20% rubber powder” is abbreviated as “20C”, and the other contrasting groups follow this rule. The high-speed shearing method was used to prepare modified asphalt. First, the base asphalt was melted at 170 °C and weighed a certain mass. Second, Nano-SiO_2_ and rubber powder mixed added into the base asphalt at 170 °C respectively, and artificially stirred for 20min at approximate 180 °C. Finally, a high-speed shear apparatus was used to grind modifiers and asphalt for 60 min at around 180 °C and with a speed of 5000 rpm.

### 2.3. Property Evaluation Tests and Aging Tests

In this study, the basic physical properties of the original asphalt without UV aging and the UV aged asphalt were tested via fully automatic asphalt softening point tester, digital ductility tester, HAILEA water bath tester manufactured by Infra Test Company of Germany (Dresden, Germany), and an automatic asphalt penetration tester manufactured by Chenxin Company of Zhejiang Province (Zhejiang, China). The Dynamic shear rheometer test of experimental samples was carried out by the dynamic shear rheometer (DSR) produced by Anton Paar Company (Graz, Austria) to study the high-temperature stability. A *Φ*25 mm parallel plate was used to conduct temperature sweep and rutting factor test with a fixed frequency equal to 10 rad/s. Complex modulus (G*) is ratio of peak stress to the peak strain in harmonic sinusoidal oscillation, which can be used as an index to express the dynamic shearing responses of asphalt. The phase angle (*δ*), complex shear modulus (G*), and rutting factor (G*/sinδ) were measured finally. The low-Temperature property was studied by using the bending beam rheometer (BBR) produced by Cannon Company (Cranberry Township, PA, USA). The loading time was equal to 240 s. Load and deformation data were collected at 8 s, 15 s, 30 s, 60 s, 120 s, and 240 s. The stiffness modulus (*S*) was measured by obtaining the displacement of asphalt beams (127 mm × 6.35 mm × 12.7 mm) at −12 °C, −18 °C, and −24 °C. Referring to a Chinese specification named as “Asphalt Rubber For Highway Engineering” (JT/T 798-2011) [37], the 180 °C Brookfield viscosity was investigate associated with rubber powder asphalt.

**Figure 1:**
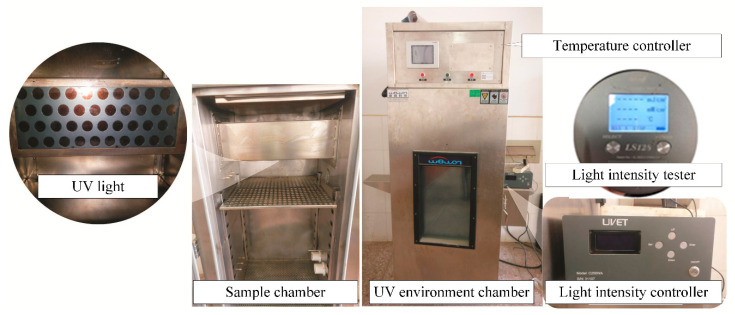
Self-made UV environment apparatus.

The UV aging test on asphalt was simulated by using a self-made UV environment apparatus (Changsha University of Science and Technology, Changsha, China). The temperature of sample chamber was controlled at 25 °C, the intensity of UV light was 14.2 mW/cm^2^. The exposed duration of samples was 7 days, which was equivalent to the UV radiation amount of three months in northwestern China known well as the high UV light intensity region. The self-made apparatus is presented in Figure 1, it consists of a UV light, a sample chamber, a light intensity controller a temperature controller, and a light intensity tester. The UV light has four rows, 10 UV-LED beads are installed in each row with filters inside to ensure the occupancy of UV light. There are cooling fans, heater, humidity and temperature testers inside the chamber. The light intensity controller can adjust the intensity of UV light and display accumulated duration, UV light intensity in the chamber can be measured by the light intensity tester. In this study, a circular disc with a diameter of 140mm is used as the asphalt sample tray, and the thickness of each binder sample is 1.5 mm. According to the formula (mass = bottom area × thickness × density), the mass of asphalt required for each sample was calculated and poured into the asphalt sample tray. After the leveling of asphalt surface, samples were cooled to a room temperature on a platform, finally samples were prepared to be exposed to the UV light via the self-made UV environment apparatus, each experimental group has five samples.

### 2.4. Evaluation Indexes of UV Aging

The UV aging degree of asphalt can be expressed by evaluation indexes, which are defined by the fluctuations of physical characteristics of testing samples. In this study, the basic properties and rheological properties of asphalt before and after UV aging were used to evaluate the UV aging resistance of asphalt, including the change of softening point (*C_sp_*), the change percent of penetration (*P_age_*), Brookfield viscosity aging index (*V_age_*), complex modulus aging index (*C_age_*), and stiffness modulus aging index (*S_age_*). All calculation formulas of indexes are expressed as follows:

(1) $$C_{\mathrm{sp}}\;=\;\mathrm{softening}\;\mathrm{point}**\;-\;\mathrm{softening}\;\mathrm{point}*$$

(2) $$P_{\mathrm{age}}\;=\;\frac{\mathrm{penetration}**}{\mathrm{penetration}*}$$

(3) $$V_{\mathrm{age}}\;=\;\frac{\mathrm{viscosity}\;\mathrm{aging}**\;-\;\mathrm{viscosity}\;\mathrm{aging}*}{\mathrm{viscosity}\;\mathrm{aging}*}$$

(4) $$C_{\mathrm{age}}\;=\;\frac{\mathrm{complex}\;\mathrm{modulus}**}{\mathrm{comples}\;\mathrm{modulus}*}$$

(5) $$S_{\mathrm{age}}\;=\;\frac{\mathrm{stiffiness}\;\mathrm{modulus}**}{\mathrm{stiffiness}\;\mathrm{modulus}*}$$

where, * means before aging, ** means after aging.

## 3. Results Discussion of Original Asphalt without UV Aging

### 3.1. Fundamental Properties

**Figure 2:**
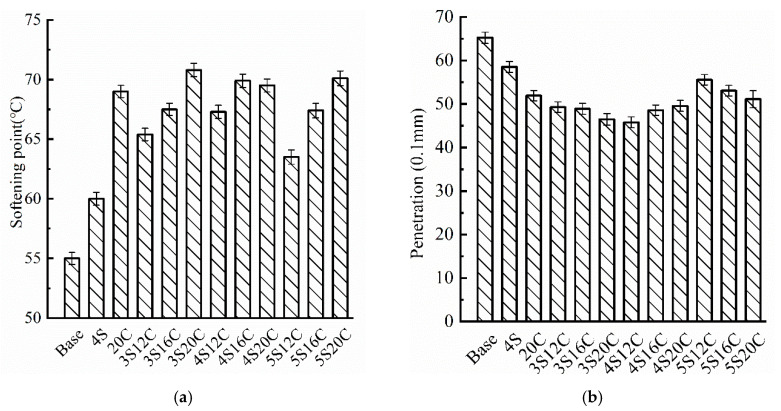
Basic physical properties testing results: (**a**) softening point, and (**b**) penetration.

From the softening point results, Figure 2 shows that the base asphalt has the smallest softening point, which might lead to the worst high-temperature performance. Whether single-doped nano-SiO_2_ or single-doped rubber powder, the softening point of the 4S and 20C increases obviously, especially the softening point of the 20C is 14° C higher than that of the base asphalt. This finding implies that nano-SiO_2_ and rubber powder have a positive effect on the high-temperature stability of asphalt. The penetration of modified asphalt at 25 °C drops compared with the base asphalt, which means that modifiers increase the consistence of the asphalt. Thus, the high-temperature stability of asphalt is improved. The penetration of 4S12C is the lowest, the value is 1.94 mm lower than the base asphalt. Compared with single-doped modified asphalt, the penetration of compound modified asphalt is lower, two types of modifiers seem to play an enhancing role in improving the consistence of asphalt.

### 3.2. Rheological Properties

#### 3.2.1. Brookfield Viscosity Test

**Figure 3:**
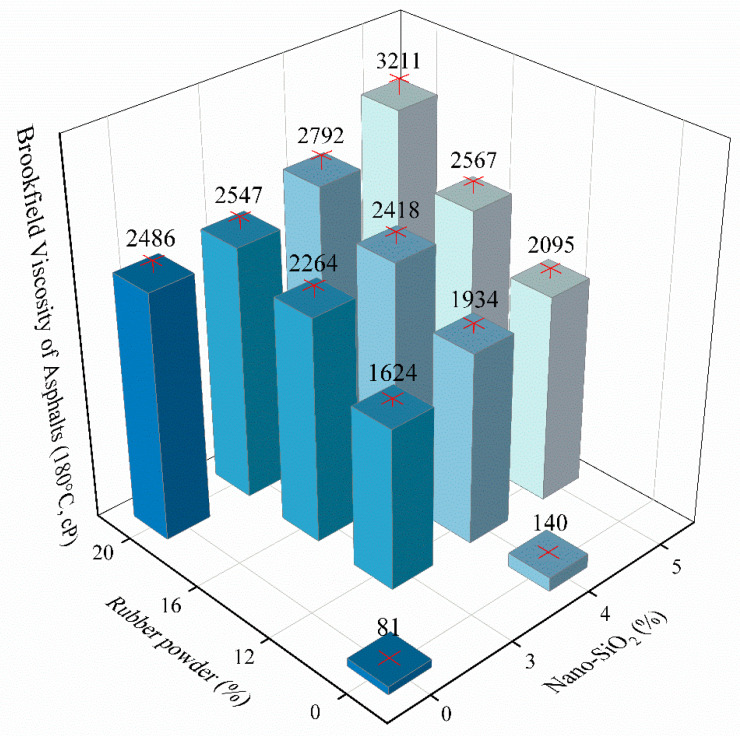
Brookfield viscosity of different experimental groups (180 °C, cP).

According to the industrial standard (JT/T 798-2011) in China, named as “Asphalt Rubber For Highway Engineering” [37], the results of Brookfield viscosity (180 °C) test are shown in Figure 3.

Viscosity of the base asphalt is the smallest, and modifiers increases the viscosity of asphalt. The incorporation of 4% nano-SiO_2_ does not increase the viscosity noticeably, because of only 59 cP increment with regard to the base asphalt. Otherwise, the incorporation of 20% rubber powder greatly increases the viscosity. The testing value rises from 81 cP to 2486 cP, because the rubber powder absorbs the asphaltene in the asphalt and forms cross-linked spatial network. Figure 3 shows that the viscosity of 5S20C is the highest among all asphalt samples, and its value is 3211 cP. However, increase of viscosity will make workability worse. With regard to compound modified asphalt, the viscosity rises with the growth of modifier content. The increment of 4S20C and 20C is larger than that of 4S and base asphalt, due to the reason that nano-SiO_2_ fills the network structure of rubber powder modified asphalt rubber. Therefore, compound modification is proved to easily strengthen the viscosity of asphalt.

#### 3.2.2. Dynamic Shear Rheometer Test

**Figure 4:**
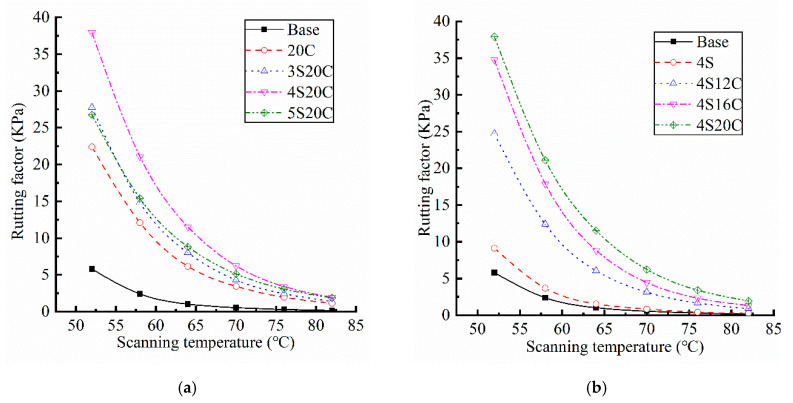
Rutting factors of asphalt: (**a**) with different nano-SiO_2_ content, and (**b**) with different rubber powder content.

According to AASHTO M320 specification, rutting factor is the ratio of complex modulus to sine of phase angle at a frequency of 10 rad/s, which can be used as an index to evaluate the high temperature performance of asphalt. Results of rutting factor for each contrasting group are listed in Figure 4, the coefficient of variation of all data is less than 10%.

Figure 4 indicates that rutting factors of the single-doped modified asphalt (4S and 20C) are higher than that of base asphalt. The rutting factor of 4S20C is the largest in the compound modified asphalt, which is nearly five time larger than that of the base asphalt. The base asphalt has the poorest high-temperature displacement resistance due to its smallest rutting factor. The incorporation of 20% rubber powder greatly improves the high-temperature stability of asphalt. With the incorporation of nano-SiO_2_, the rutting factor of asphalt shows a trend of increase first and then decrease. When the content of nano-SiO_2_ is 4% in Figure 4a, the rutting factor reaches the maximum. Figure 4b shows that the rutting factor exhibits a very obvious improvement with the incorporation of rubber powder.

#### 3.2.3. Bending Beam Rheometer Test

**Figure 5:**
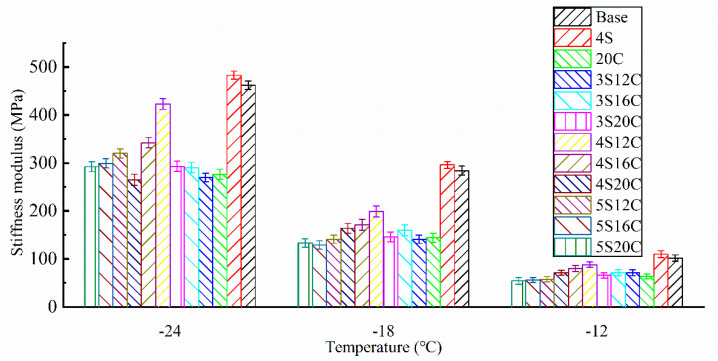
Stiffness modulus *(S)* of asphalt at different temperatures.

Stiffness modulus *(S)* of each group can be seen in Figure 5. The stiffness modulus increases as the test temperature decreases, the creep rate decreases as the test temperature decreases. This fact means that the asphalt becomes hard and brittle as the temperature decreases, and brittle cracking is likely to occur under external force.

The stiffness modulus of 4S is the highest and even slightly higher than that of base asphalt, which presents that the addition of 4% nano-SiO_2_ makes asphalt stiffer and reduces the low-temperature cracking resistance of asphalt. The stiffness modulus of 20C is the lowest, because 20% rubber powder increases the elasticity of asphalt at low temperature. Consequently, rubber powder greatly improves the low-temperature property of asphalt. By keeping the content of nano-SiO_2_ the same, the stiffness modulus decreases with the increase of rubber powder content. By keeping the content of rubber powder the same, the largest stiffness modulus can be easily seen in 4% nano-SiO_2_. Thus, it is essential to solve the degradation on the low-temperature property of 4S, 4S20C is the optimum compound ratio due to its smallest stiffness modulus among 4S12C, 4S16C, and 4S20C. This finding could be related to the network structure of rubber powder modified asphalt. 4% nano-SiO_2_ seems to be a suitable dosage, which can be used as tiny particles to fill the defects in the network structure and make rubber powder modified asphalt well-distributed.

## 4. Result Discussion of UV Aging Asphalt

### 4.1. Evaluation Indexes of Basic Properties

**Figure 6:**
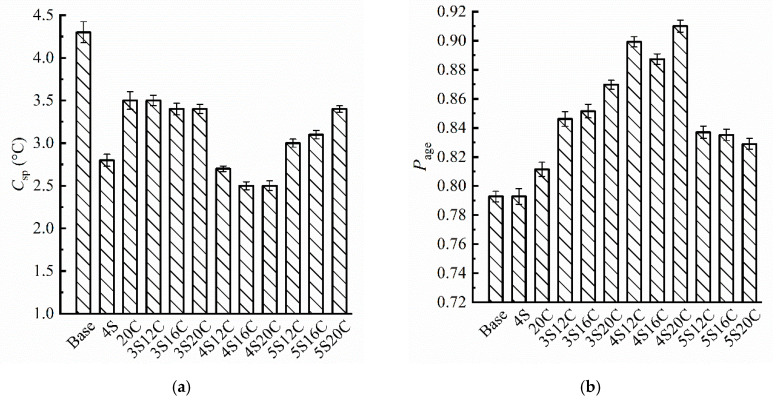
Evaluation indexes of basic properties: (**a**) the change of softening point (*C_sp_*), and (**b**) the change percent of penetration (*P_age_*).

From Figure 6a, b it can be seen that the *C_sp_* of modified asphalt is less than that of base asphalt, and the *P_age_* of modified asphalt is greater than that of base asphalt. Results show that the modifiers enhance the UV aging resistance of asphalt and improved the durability of asphalt. 20C indicates that the rubber powder can improve the anti-UV aging of asphalt. It is due to the fact that a small amount of carbon black in rubber powder enters into asphalt during the shear to prepare rubber powder modified asphalt, and carbon black is known to absorb UV light slightly.

However, the increase of rubber powder content does not significantly improve the UV aging resistance of modified asphalt when the nano-SiO_2_ content is the same. Moreover, the protection effect of asphalt on the UV-aging is more sensitive to nano-SiO_2_, because of reflection of nano-SiO_2_ to the UV light. When the content of nano-SiO_2_ is 5%, the UV-aging resistance of the compound modified asphalt is deteriorated, excessive agglomeration of nano-SiO_2_ affects the modification. In all group, 4S20C ranks as the best dosage to have the smallest *C_sp_* and the largest *P_age_*, which depicts 4S20C presents the strongest resistance to UV aging on softening point and penetration.

### 4.2. Evaluation Indexes of Rheological Properties

#### 4.2.1. Brookfield Viscosity Aging Index

**Figure 7:**
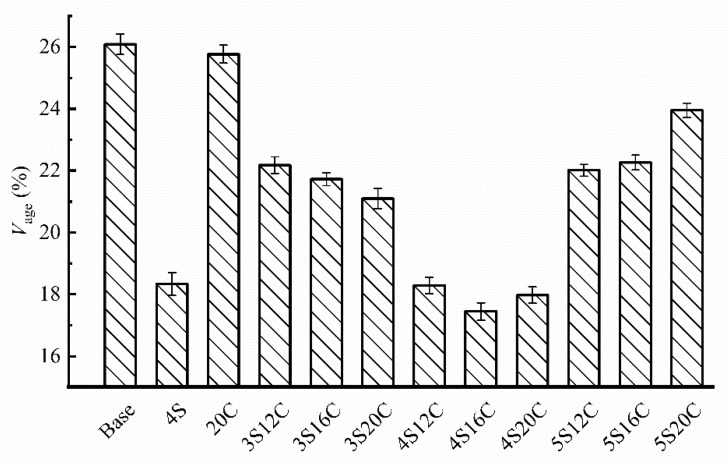
Brookfield viscosity aging index (*V_age_*) with different modifier content.

Figure 7 shows that minor anti-UV aging effect can be seen in rubber powder modified asphalt (20C) due to the carbon black, because the *V_age_* of 20C is slightly lower than that of base asphalt. The *V_age_* of nano-SiO_2_ modified asphalt decreases sharply with respect to other groups, this finding indicates that nano-SiO_2_ can effectively improve the anti-UV aging property of asphalt. However, the *V_age_* starts to rise when nano-SiO_2_ content reaches 5%. Additionally, the *V_age_* of 4S16C is the lowest, and the *V_age_* of 4S20C is very close to that of 4S16C.

#### 4.2.2. Complex Modulus Aging Index

**Figure 8:**
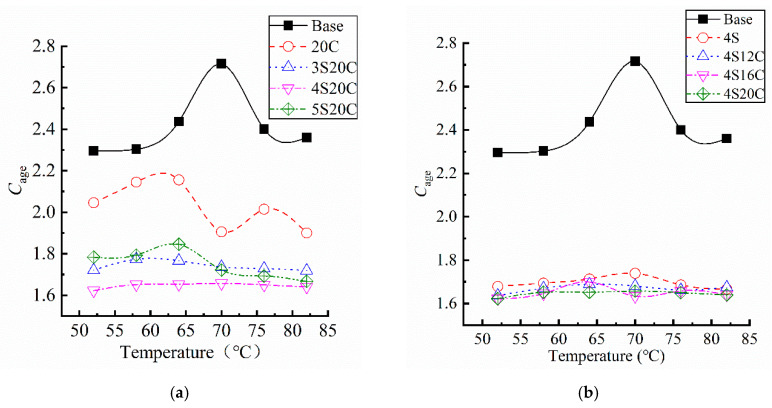
Complex modulus aging index (*C_age_*) of asphalt samples: (**a**) with different nano-SiO_2_, and (**b**) with different rubber powder.

The temperature sweep of each asphalt after UV aging was scanned at 10rad/s and within linear viscoelastic domain, and the *C_age_* is shown in Figure 8, the coefficient of variation of all data is less than 10%. In Figure 8, obvious differences can be summarized between the base asphalt and modified asphalt. Otherwise, the influence of temperature on the *C_age_* seems to be random, especially at 70 °C.

The *C_age_* of modified asphalt is lower than that of the base asphalt, which proves that the incorporation of nano-SiO_2_ and rubber powder improves the UV aging resistance of asphalt. It can be seen from Figure 8a that the *C_age_* curve of the rubber powder modified asphalt is located below that of the base asphalt, rubber powder has certain effect on the improvement of UV aging resistance. With the influence of nano-SiO_2_, the *C_age_* decreases significantly. When the content of nano-SiO_2_ is 4%, the *C_age_* is the lowest, and the anti-UV aging resistance is enhanced to a large degree. Figure 8b presents that the *C_age_* of compound modified asphalt behaves almost the same. It proves that nano-SiO_2_ is the main component to play anti-UV aging role.

#### 4.2.3. Stiffness Modulus Aging Index

**Figure 9:**
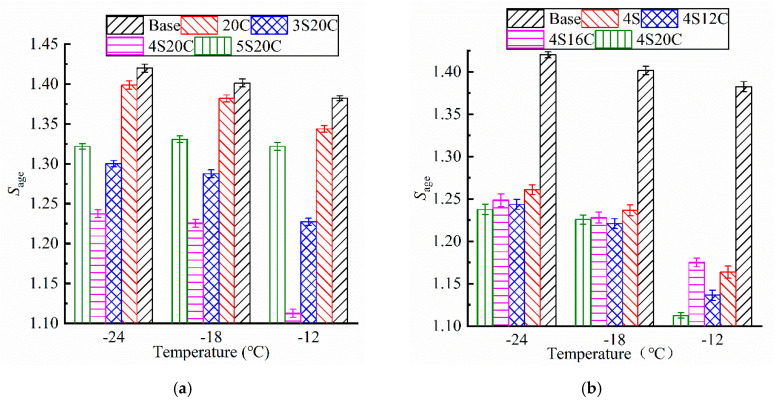
Stiffness modulus aging index (*S*_age_) of asphalt with different modifier content: (**a**) with different nano-SiO_2_, (**b**) with different rubber.

As shown in Figure 9, the *S_age_* of all testing groups subjected to UV aging is greater than 1. Asphalt becomes brittle and hard as the stiffness modulus becomes larger, and the low-temperature cracking will be more likely to occur on asphalt pavement. The *S_age_* of modified asphalt is smaller than that of the base asphalt, which implies that nano-SiO_2_ and rubber powder can protect the UV aging degradation on the low-temperature property of asphalt. In Figure 9a, the *S_age_* of nano-SiO_2_ modified asphalt present obvious decrease than that of the base asphalt and 20C. Figure 9b expresses that the *S_age_* of 4S12C, 4S16C, and 4S20C almost behave the same at −24 °C and −18 °C, which directs that the rubber powder is not sensitive to stiffness modulus increase due to UV aging. It is obvious that 4S20C must be chosen as the best compound ratio related to the lowest increase of stiffness modulus.

## 5. Conclusions

Property evaluation of the non-aged asphalt indicates that the compound modification improves the high-temperature stability of asphalt significantly, and the effect of compound modification becomes more obvious than the single-doped modification. Nano-SiO_2_ plays an adverse role on the low-temperature cracking of asphalt, because of the slightly increase of stiffness modulus. Contrarily, rubber powder increases the elasticity and decreases the stiffness modulus of asphalt at low temperatures. Therefore, the compound modification might solve the low-temperature cracking issue due to the nano-SiO_2_. Additionally, 4% nano-SiO_2_ and 20% rubber powder compound modified asphalt (4S20C) presents the best performance in this study.

By directly exposing to UV aging, compound modification is proved to improve the anti-UV aging performance of asphalt. As the dosage of nano-SiO_2_ changes, the aging degree of asphalt fluctuates significantly. However, the effect of rubber powder on anti-UV aging is concluded to be less sensitive. Nano-SiO_2_ performs a predominant role on enhancing the UV aging resistance. Moreover, 4% nano-SiO_2_ and 20% rubber powder compound modified asphalt (4S20C) is testified to be the optimum dosage in this study, due to its improvement on the UV aging resistance and elimination of the low-temperature property degradation caused by nano-SiO_2._

## Figures and Tables

**Table 1:** Physical properties of nano-SiO_2._

Items	Unit	Testing Result
Outer diameter	nm	10~20
Purity	weight, %	>99.8
Molecular weight	—	60.08
PH	—	5~7
Tap density	m^2^/g	250

**Table 2:** Physical properties of 80# mesh waste rubber powder (%).

Items	Passing Rate	Water Content	Ash Content	Metal Content	Carbon Black Content	Rubber Hydrocarbon
Testing results	97	0.72	7	0.004	30	34
Limit	>96	<1	<8	<0.01	>28	>30
Specification code	GB/T1 9208-2008	GB/T1 9208-2008	GB/T4 498-1997	GB/T1 9208-2008	GB/T1 4837-1993	GB/T1 4837-1993

**Table 3:** Physical properties of 70#A asphalt.

Items	Technical Limit	Test Result	Specification Code
Penetration (25 °C, 100 g, 5 s), 0.1mm	60~80	65.2	T0604
Penetration index	−1.5~+1.0	0.73	T0604
Softening point, °C	≥46	48	T0606
Density (15 °C), g/cm^3^	—	1.034	T0603
Ductility (15 °C, 5 cm/min), cm	≥100	>100	T0605
Wax content, %	≤2.2	2	T0615
Dynamic viscosity (60 °C), Pa·s	≥180	215	T0620
